# Nanomaterials for Sustainable Green Energy

**DOI:** 10.3390/nano16060373

**Published:** 2026-03-20

**Authors:** Zhao Ding, Liangjuan Gao

**Affiliations:** College of Materials Science and Engineering, Chongqing University, Chongqing 400044, China

The ongoing transition toward sustainable energy systems is increasingly driven by advances in materials science. As global demand for clean energy continues to rise, the development of materials capable of enabling efficient energy conversion, storage, and utilization has become a central research priority. Among the wide range of materials explored in recent years, nanomaterials have attracted particular attention. Their high surface areas, tunable electronic structures, and rich interfacial chemistry provide opportunities to regulate catalytic processes and mass transport at the nanoscale, thereby offering new solutions to several persistent challenges in energy technologies. This Special Issue, “Nanomaterials for Sustainable Green Energy”, brings together eleven contributions that highlight recent progress in nanomaterials for energy-related applications. The collection includes two review articles and nine research papers addressing hydrogen-related materials, electrocatalysis for renewable electricity conversion, carbon-based energy materials, and sustainable synthesis strategies. Although these studies cover a broad range of topics, they can be viewed within three closely related areas: hydrogen energy materials, electrocatalysis for renewable electricity conversion, and carbon-neutral materials and circular carbon technologies. Together, they illustrate how nanoscale materials design is contributing to several key aspects of the emerging sustainable energy landscape.

The issue opens with hydrogen-related materials, reflecting the continued importance of hydrogen as a potential energy carrier in low-carbon energy systems [1,2]. The review by Xu et al. (contribution 1) provides an overview of recent developments in nanomaterials for solid-state hydrogen storage. Particular attention is given to nanoscale structural regulation, interface engineering, and catalytic modification strategies that can improve hydrogen sorption thermodynamics and kinetics. By linking materials design with the fundamental limitations of hydride systems, the review offers a useful framework for current research in hydrogen storage materials. Two subsequent studies focus on Mg-based hydrogen storage systems from complementary experimental and theoretical perspectives. Huang et al. (contribution 2) demonstrate that the combination of WS_2_ nanotubes with Pd can significantly enhance hydrogen sorption in AZ31 magnesium alloys through a synergistic catalytic effect. In parallel, Chrafih et al. (contribution 3) employ a multiscale modeling approach to examine doped MgH_2_ nanomaterials, integrating density functional theory calculations with reactor-scale simulations. Their results highlight how nanoscale compositional modifications can influence hydrogenation performance under practical operating conditions. Taken together, these studies illustrate the value of combining experimental materials design with predictive modeling in the development of hydrogen storage systems. Hydrogen-related research in this issue also extends to production pathways. Cao et al. (contribution 4) investigate catalytic methane decomposition using FeCo alloy nanoparticles generated in situ from layered double hydroxides. The process enables efficient methane conversion while simultaneously producing hydrogen and carbon nanofibers, demonstrating a route that links hydrogen production with the generation of value-added carbon materials.

A second group of contributions addresses electrocatalysis for renewable electricity conversion, particularly oxygen-related electrochemical reactions that underpin technologies such as water electrolysis [3], fuel cells [4], and metal–air batteries [5]. Yan et al. (contribution 5) review recent advances in low-dimensional carbon nanomaterials—including carbon dots, carbon nanotubes, and graphene-based structures—for oxygen electrocatalysis. The review highlights how heteroatom doping, defect engineering, and electronic structure modulation can be used to regulate catalytic activity. Three research papers then explore different strategies for improving oxygen evolution catalysis. Zhang et al. (contribution 6) report RuO_2_–Co_3_O_4_ composites that exhibit enhanced oxygen evolution performance, emphasizing the role of compositional synergy in mixed oxide catalysts. Chen et al. (contribution 7) demonstrate that nickel oxide thin films produced by atomic layer deposition can serve as efficient oxygen evolution catalysts, illustrating the advantages of precise nanoscale structural control. In another approach, Ahmed et al. (contribution 8) show that catalytic activity can emerge dynamically under operating conditions through the in situ formation of a CoOOH@Co_3_S_4_ heterostructure. Considered together, these studies reflect several complementary approaches to electrocatalyst design, including compositional tuning, controlled nanoscale fabrication, and structural evolution during operation.

The final group of papers addresses carbon-neutral materials and circular carbon technologies, an area receiving increasing attention as strategies for carbon utilization continue to develop [6]. Cheng et al. (contribution 9) investigate the electrochemical reduction in CO_2_ in molten salt systems and demonstrate that electrolyte composition plays a key role in determining the morphology of the resulting carbon nanostructures. Their results suggest that selective formation of nanotubes, nanospheres, or mixed carbon structures can be achieved by adjusting molten salt chemistry. Dong et al. (contribution 10) approach carbon materials from a different perspective, reporting the microwave-assisted synthesis of hierarchical porous carbon aerogels derived from food waste. The resulting materials exhibit promising electrochemical performance as supercapacitor electrodes, highlighting the potential of biomass-derived precursors for advanced carbon materials. The issue concludes with a study by Xu et al. (contribution 11), who describe a solvent-controlled synthesis of monodisperse CeO_2_ octahedra together with successful recycling of the reaction medium. Although focused primarily on synthetic methodology, this work emphasizes an important aspect of sustainable nanomaterials research—the development of greener and more resource-efficient preparation routes.

Taken together, the contributions collected in this Special Issue demonstrate the broad impact of nanomaterials in sustainable energy research. From hydrogen storage and production to electrocatalysis, carbon conversion, and sustainable synthesis, the studies presented here highlight how nanoscale control over composition, structure, and interfaces can open new opportunities for designing more efficient energy materials and processes. The editors hope that this collection will serve as a useful reference for researchers working at the intersection of nanomaterials science and sustainable energy technologies and will stimulate further progress in this rapidly evolving field.
